# The Putative *Leishmania* Telomerase RNA (*Leish*TER) Undergoes *Trans*-Splicing and Contains a Conserved Template Sequence

**DOI:** 10.1371/journal.pone.0112061

**Published:** 2014-11-12

**Authors:** Elton J. R. Vasconcelos, Vinícius S. Nunes, Marcelo S. da Silva, Marcela Segatto, Peter J. Myler, Maria Isabel N. Cano

**Affiliations:** 1 Seattle Biomedical Research Institute, Seattle, Washington, United States of America; 2 Departamento de Genética, Instituto de Biociências, Universidade Estadual Paulista (UNESP), Botucatu, São Paulo, Brazil; 3 Universidade Estadual deCampinas (UNICAMP), Campinas, São Paulo, Brazil; 4 Department of Global Health, University of Washington, Seattle, Washington, United States of America; 5 Department of Biomedical Informatics and Medical Education, University of Washington, Seattle, Washington, United States of America; Rutgers New Jersey Medical School, United States of America

## Abstract

Telomerase RNAs (TERs) are highly divergent between species, varying in size and sequence composition. Here, we identify a candidate for the telomerase RNA component of *Leishmania* genus, which includes species that cause leishmaniasis, a neglected tropical disease. Merging a thorough computational screening combined with RNA-seq evidence, we mapped a non-coding RNA gene localized in a syntenic locus on chromosome 25 of five *Leishmania* species that shares partial synteny with both *Trypanosoma brucei* TER locus and a putative TER candidate-containing locus of *Crithidia fasciculata*. Using target-driven molecular biology approaches, we detected a ∼2,100 nt transcript (*Leish*TER) that contains a 5′ spliced leader (SL) cap, a putative 3′ polyA tail and a predicted C/D box snoRNA domain. *Leish*TER is expressed at similar levels in the logarithmic and stationary growth phases of promastigote forms. A 5′SL capped *Leish*TER co-immunoprecipitated and co-localized with the telomerase protein component (TERT) in a cell cycle-dependent manner. Prediction of its secondary structure strongly suggests the existence of a *bona fide* single-stranded template sequence and a conserved C[U/C]GUCA motif-containing helix II, representing the template boundary element. This study paves the way for further investigations on the biogenesis of parasite TERT ribonucleoproteins (RNPs) and its role in parasite telomere biology.

## Introduction

*Leishmania* spp. are trypanosomatid protozoa, considered ancient eukaryotes whose nuclear genome is organized in linear chromosomes [1], [2]. Like most eukaryotes, their chromosomes end termini are characterized by sequences known as telomeres [3]. *Leishmania* telomeres are formed by conserved 5′-TTAGGG-3′ telomeric repeats that typically end in a 3′ overhang that serves as substrate for repeat addition by telomerase [3]–[6], a ribonucleoprotein enzyme minimally composed of two catalytically essential subunits: the telomerase reverse transcriptase protein (TERT) and the telomerase RNA (TER) component, which contains the template that specifies the sequence of the telomeric repeats [7], [8]. Enzyme activity has been detected in cell extracts of promastigotes of three *Leishmania* species, and also from different *Trypanosoma brucei* and *Trypanosoma cruzi* replicative stages [9]–[11]. The trypanosomatids' TERT component is one of the largest telomerase (MW ∼156 kDa) described so far and the TERT enzyme from *Leishmania* species shows greater sequence similarity (86–95%) with each other than with the telomerases of other eukaryotes [12], [13]. Although trypanosomatid TERT contains some important amino acid substitutions within the conserved TERT motifs, the *Leishmania* and *Trypanosoma* TERT components present all the conserved structural features shared with other TERTs, such as the N-terminus motifs that are essential for telomerase RNA (TER) binding and enzyme activity [14]–[16], the central domain that contains a less conserved telomerase-specific T motif, the reverse transcriptase motifs that are essential for enzyme activity, and a less conserved C-terminal domain [12], [17]–[20]. Biochemically, *Leishmania* and *Trypanosoma* TERTs resemble other telomerases, as they are able to add TTAGGG repeats to the 3′ end of the G-rich telomeric strand and fulfill other essential criteria for telomerase activity, such as RNase A sensitivity. Enzyme processivity differs among trypanosomatids, with *T. brucei* TERT being the most processive compared to *T. cruzi* and *Leishmania* TERTs [9]–[11]. The purification of *Leishmania amazonensis* TERT enzyme was only achieved using semi-purified protein extracts fractionated on a G-rich telomeric DNA affinity column (G-DNA), indicating that similar to *T. cruzi* telomerase, *Leishmania* TERT bound tightly to an antisense 2′*O*-methyl oligonucleotide complementary to the *T. brucei* TER template sequence [10], [11]. This was the first hint that the *Leishmania* TER (*Leish*TER) template sequence was similar to the predicted minimum model for the RNA template region used by *T. brucei* telomerase [9].

Recently, with the characterization of the telomerase RNA molecule of *T. brucei* (*Tb*TER), it was possible to confirm that the *Tb*TER template sequence 5′-CCCTAACCCTA-3′ differs from the previously predicted template (5′-CCCTAACCC-3′) only by having two nucleotides more at its 3′ end [9], [21]. In addition, *Tb*TER is processed through *trans*-splicing and was shown to interact and to copurify with TbTERT *in vivo*. Deletion or silencing of *TbTER* causes progressive shortening of telomeres and mutations in its template domain results in corresponding mutant telomere sequences, demonstrating that in *T. brucei* it is essential for telomerase activity [21], [22]. Moreover, like TERs from different eukaryotes, *Tb*TER differs greatly in nucleotide sequence and size [21], [22]. It shares some secondary structural elements conserved in most, but not all, eukaryotic TERs, such as a putative pseudoknot domain, that includes at the 5′ end the TER template motif, in addition to helix I and helix II, the latter of which contains a putative template boundary element (TBE) [23]. *Tb*TER also binds the core proteins of the C/D small nucleolar RNA (snoRNA) family and associates with the methyltransferase-associated protein, whose homolog also binds to mammalian TER [22].

Here, we report the identification and characterization of the putative TER components from two *Leishmania* species, *Leishmania major* (*Lm*TER) and *Leishmania amazonensis* (*La*TER), using *in silico* and experimental approaches. *Leishmania* TERs (*Leish*TER), like *Tb*TER, appear to be transcribed by RNA pol II, as they are located in the sense orientation within an mRNA directional gene cluster (DGC), and the putative mature RNA was amplified using the 39 nt spliced leader (SL) RNA sequence commonly positioned at the 5′ end of most mature trypanosomatids RNA pol II transcribed RNAs. The 3′ end of both *Leish*TERs was also amplified by 3′ RAcE using oligo-dT and total RNA, resembling the mature form of *Tb*TER [21]. *Leish*TERs also contain a template domain that is almost identical to *Tb*TER template sequence. Using immunofluorescence coupled with RNA *in situ* hybridization, we demonstrated that *Lm*TER co-localizes with LmTERT in parasite nucleus. In addition, a 5′SL capped *Lm*TER immunoprecipitates with the telomerase reverse transcriptase component, suggesting that the mature transcript is part of the ribonucleoprotein complex. We predicted a partial secondary structure of *Leishmania* TER that shows some conserved structural features shared among its *Tb*TER counterpart and other TERs.

Further experiments are required to elucidate the biological importance of the *Leish*TER molecule for parasite survival and, thus, whether it will serve as a target for anti-parasite therapy. This discovery would be of great significance because the genus *Leishmania* comprises several species that cause leishmaniasis, which are neglected tropical diseases that threaten hundreds of millions of people around the world but lack treatment options, vaccines and prophylaxis protocols (World Health Organization 2010).

## Materials and Methods

### *in silico* analyses of *LeishTER* candidates

We started the *in silico* screening for the *Leishmania* TER gene performing a sensitive BLASTn search [24] (-FF –W7 –m8) against *L. major, L. infantum, L. braziliensis, L. mexicana* and *L. tarentolae* genomes (TriTrypDB 6.0) using as query the template sequence that consisted of two tandem telomeric hexamer repeats (THR) identified previously ([6] and Cano, MIN, Personal Communication). Since the telomeric 3′ overhang may vary between *Leishmania* species [6], [25], we used all six iterations of two copies of telomeric hexamer repeats (THRs) as follow: query_1: 5′ CCTAACCCTAAC 3′; query_2: 5′ CTAACCCTAACC 3′; query_3: 5′ CCCTAACCCTAA 3′; query_4: 5′ ACCCTAACCCTA 3′; query_5: 5′ AACCCTAACCCT 3′; query_6: 5′ TAACCCTAACCC 3′.

A PERL script was written to discard the great number of BLAST hits falling within the telomeres, which must have a length defined by the user. We arbitrarily chose a value of 9 kb from both chromosome ends to filter out the BLAST hits within those regions in each chromosome from all species. The template query_4 was the only one presenting a perfect match in a syntenic locus in all *Leishmania* genomes analyzed, and this region was subjected to a deeper analysis on a comparison to other trypanosomatids (See results, Table 1 and Table 2).

**Table 1:** All extra-telomere BLASTn hits for six different combinations of the 12 nt TER template sequence in the *L. major* genome.

Query ID	Subject ID	Identity %	Alignment length	Mismatches	Gap Openings	q. start	q. end	s. start	s. end	E-value	Bit score
query_1	LmjF.28	100	12	0	0	1	12	544272	544283	3.2	24.3
query_2	LmjF.28	100	12	0	0	1	12	544273	544284	3.2	24.3
query_3	LmjF.28	100	12	0	0	1	12	544271	544282	3.2	24.3
**query_4**	**LmjF.25**	**100**	**12**	**0**	**0**	**1**	**12**	**333352**	**333363**	**3.2**	**24.3**
query_5	LmjF.28	100	12	0	0	1	12	544275	544286	3.2	24.3
query_6	LmjF.28	100	12	0	0	1	12	544274	544285	3.2	24.3

**Table 2:** Extra-telomere BLASTn hits of the TER template sequence on chromosome 25 from the other *Leishmania* species: *L. infantum* (LinJ), *L. mexicana* (LmxM), *L. tarentolae* (LtaP) and *L. braziliensis* (LbrM).

Query ID	Subject ID	Identity %	Alignment length	Mismatches	Gap Openings	q. start	q. end	s. start	s. end	E-value	Bit score
query_4	LinJ.25	100	12	0	0	1	12	322179	322190	3.2	24.3
query_4	LmxM.25	100	12	0	0	1	12	322685	322696	3.1	24.3
query_4	LtaP25	100	12	0	0	1	12	329780	329791	3.1	24.3
query_4	LbrM.25	100	12	0	0	1	12	277119	277130	3.1	24.3

All the depicted loci are syntenic to the one in *L. major* chromosome 25 shown in Table 1.

**Figure 1:**
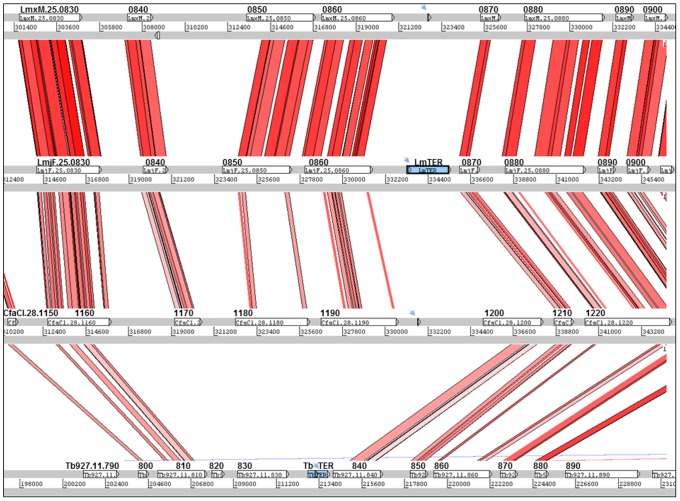
Protein-coding gene synteny view at the TER locus of trypanosomatid species from three different genera. tBLASTx comparisons were performed, followed by a visualization of the results using the ACT tool. Gray bars represent the forward and reverse strands of DNA, and the numbers between them correspond to the absolute coordinates within the chromosome where this syntenic locus is located: *L. mexicana* (LmxM) and *L. major* (LmjF) chromosome 25, *C. fasciculata* (CfaCl) chromosome 28 and *T. brucei* (Tb927) chromosome 11. The reddish-pink lines between sequences represent sequence similarity from tBLASTx analyses. The white box features correspond to CDSs, and the blue ones indicate the TER gene of both *L. major* and *T. brucei*. Blue arrows point to the conserved 12 nt TER-template sequence (5′ ACCCTAACCCTA 3′) found in all species at this particular locus (in *T. brucei* it is 11 nt, 5′ CCCTAACCCTA 3′). TriTrypDB gene accession numbers are written inside each white box and the last digits placed above the boxes. The two protein-coding genes immediately upstream of *TbTER* (*Tb927.11.820* and *830*) appear to be *Trypanosoma-*specific, whereas *LmjF.25.0840*, *0850* and *0860* are indicative of *Leishmania-*/*Crithidia*-specific genes. Despite the synteny disruption observed on the protein-coding content nearby the TER gene, this non-coding RNA seemed to be retained by evolutionary pressure on the same syntenic position in the three distinct genera.

We ran tBLASTx [24] from the blast+ package and used its output as an input to the ACT tool [26] to compare the synteny regarding the TER locus among *L. major* and *L. mexicana* chr25, *Trypanosoma brucei* chr11 and *Crithidia fasciculata* chr28 (see results, Figure 1).

ClustalW [27] and Jalview [28] were used for the generation and visualization of global multiple alignments, respectively. The RNA secondary structures were determined by first running RNAalifold [29], using as input the.aln ClustalW file containing the expected TER sequences for all five *Leishmania* species analyzed, and then the constraints from the consensus structure were applied to the individual TER modeling by the execution of mfold with default parameters [30]. Pknots [31], KnotSeeker [32], and Turbofold [33] algorithms were applied without any success in our attempt to detect pseudoknots along the entire *Leishmania* TER sequences.

### Cell lines and cell culture

Promastigotes forms of *L. major* LT252 (MHOM/IR/1983/IR) and *L. amazonensis* (MHOM/BR/73/M2269) were cultured at 26°C in M199 medium supplemented with 10% heat-inactivated fetal bovine serum as previously described [34].

### *L. major* and *L. amazonensis* total RNA isolation

Total RNA from *L. major* and from *L. amazonensis* promastigotes and that obtained from nuclear protein extracts and IP isolates were isolated using TRIzol reagent (Invitrogen) according to the manufacturer's instructions. Extracted RNAs were solubilized with 50 µL of water and treated with RNase free-DNase I (Life Technologies) in 1X DNase I buffer (10 mM Tris-HCl, 2.5 mM MgCl_2_, 0.5 mM CaCl_2_, pH 7.6) for 15 minutes at room temperature. The reaction was inactivated by the addition of 1 µl 25 mM EDTA solution and heating for 10 min at 65°C.

### Northern blot analyses

Total RNA isolated from *L. major* and from *L. amazonensis* promastigotes was fractionated in a 1.5% agarose/2.0 M formaldehyde gel electrophoresis. A 347 bp *Lm*TER fragment generated by PCR using the e+f primers (Table S1) was labeled with α-dGTP [^32^P] and used as the specific probe. A membrane containing transferred RNAs was pre-hybridized with solution I (2X SSC, 0.5% SDS, 0.1% Ficoll, 0.1%PVP, 0.1% BSA, 0.1 mg/mL ssDNA) and hybridized with *Lm*TER probe in solution II (2X SSC, 0.5% SDS, 0.02% Ficoll, 0.02% BSA, 0.1 mg/mL ssDNA). The membrane was washed twice with wash solution I (2x SSC, 0.1% SDS) at room temperature, once with wash solution II (1x SSC, 0.1% SDS) at room temperature, once with wash solution II at 65°C and finally twice with wash solution III (0.2X SSC, 0.1% SDS) at 65°C. The membrane was exposed up to 7 days at −80°C.

### 5′ and 3′ Rapid Amplification of cDNA Ends (5′ and 3′ RAcE)

To map both the 5′ and the 3′ends of the *Lm*TER transcript, cDNA was synthesized from total RNA (1.5 µg) using the QIAGEN OneStep RT-PCR Kit and using respectively the combination of primers a+d [Forward - spliced leader sequence (5' RAcE) and Reverse - (5' RAcE)] and g+h [Forward (3′RAcE) and Reverse - oligo dT (3′RAcE)] (for primer sequences and names see Table S1). Thermal cycler conditions for 5′ RAcE were 30 minutes at 50°C for reverse transcription, 15 minutes at 95°C for the initial PCR activation step and 40 cycles of denaturation for 45 seconds at 95°C, annealing for 1 minute at 57°C and extension for 1 minute at 72°C. Thermal cycler conditions for 3′ RAcE were 30 minutes at 50°C for reverse transcription, 15 minutes at 95°C for the initial PCR activation step and 60 cycles of denaturation for 45 seconds at 95°C, annealing for 1 minute at 58°C and extension for 1 minute at 72°C.

### Cloning and characterization of 5′ and 3′ RAcE products

The 5′ and 3′ RAcE products were purified from agarose gel using the Wizard SV Gel and PCR Clean-Up System (Promega) and then inserted into the TOPO TA Cloning vector (Invitrogen). The resulting plasmids were analyzed by *EcoR*I restriction enzyme digestion, and positive clones were sequenced with M13 primers. The sequences generated were aligned against the *Lm*TER predicted sequence.

### Indirect immunofluorescence (IIF)

Exponentially growing promastigote cells were washed with 1X PBS (137 mM NaCl, 2.7 mM KCl, 10 mM Na_2_HPO_4_ and 2 mM KH_2_PO_4_) and fixed in 1% (v/v) formaldehyde in 1X PBS for 5 min at room temperature. Cells were then treated with 0.1% Triton-X 100 in 1X PBS for 10 min and free aldehyde molecules were neutralized with 0.1 M glycine in 1X PBS for 10 min at room temperature. Cells that were treated with RNase A (Invitrogen) were washed with 1X PBS and then incubated with 20 µg of RNase A at 37°C for 30 min. Cells not treated with RNase A were washed with 1X PBS and incubated with 1X PBS at 37°C for 30 min. RNase A-treated and -non-treated cells were washed with 1X PBS and incubated with rabbit anti-LaTERT serum, obtained from recombinant *Leishmania amazonensis* TERT N-terminal region containing a putative telomerase RNA binding domain (TRD) (Giardini & Cano, unpublished data); LaTERT and LmTERT share about 95% identity (LmTERT) [12]). α-LaTERT was diluted (1∶2000) in blocking solution (4% (w/v) bovine serum albumin) for 12 h at 4°C. Goat anti-rabbit IgG (2 mg/mL) labeled with Alexa Fluor 555 (Invitrogen) was diluted 1∶3000 and used as the secondary antibody. Cells were deposited on poly-L-lysine coated slides and used in fluorescence RNA *in situ* hybridization assays.

### Indirect immunofluorescence coupled with RNA *in situ* hybridization

Poly-L-lysine-coated slides containing RNase A-treated and non-treated log-phase promastigote cells were dehydrated using an ethanol series (70%, 80% and 90%) and incubated with 0.3 µg.mL^−1^ of TelG-FITC PNA probe (Panagene) diluted in 1X hybridization buffer (70% formamide, 20 mM Tris-HCl, pH 7.0 and 1% BSA) at 4°C for 12 h in the dark using a 25 µl frame (Gene Frame, Pierce Biotechnology). Thereafter, the slides were washed with 1X washing buffer (50 mM Tris-HCl, pH 7.6) and dehydrated again using 70%, 80% and 90% ethanol. VECTASHIELD Mounting Medium with DAPI (Vector Labs) was used as anti-fade mounting solution and to stain nuclear and kinetoplast DNA. Finally, slides were sealed using coverslips. For these experiments, images were analyzed with a Nikon 80i fluorescence microscope and captured with a digital camera (DS-Fi1, Nikon). When necessary, images were superimposed using NIS elements software (version Ar 3.10). The parasites cultures used for the FISH-IF analysis were not synchronized since we were able to morphologically discriminate *L. major* promastigote cell cycle phases based on a previous report [35].

### Immunoprecipitation (IP) and western blot analyses

Two hundred micograms of nuclear protein extract obtained according to [9] was used as input in IP assays, in conjunction with 10 µg of rabbit anti-LaTERT serum or 10 µg of the corresponding pre-immune serum as control. The IP assays were performed using Dynabeads Protein A (Novex by Life Technologies) according to the manufacturer′s instructions. At the end of the assay, one-tenth of each IP eluate and 10% of the input were fractionated by 12% SDS-PAGE and transferred to nitrocellulose membranes (Bio-Rad) in transfer buffer (48 mM Tris-HCl, pH 8.3, 39 mM glycine, 20% methanol (v/v)) at 16°C. The membranes were probed with mouse anti-LaTERT and rabbit anti-LmNOP1 (control) used as primary antibodies. Goat anti-rabbit IgG (H+L) and goat anti-mouse IgG (H+L) HRP-conjugates (Bio-Rad) were used as secondary antibodies. The reactions were revealed using the ECL western blotting analysis system (GE Healthcare) according to the manufacturer's instructions.

### Amplification of *Lm*TER from protein extracts and from IP eluates

Total RNA from protein extract (input) and IP eluates were obtained using TRIzol reagent (Invitrogen) as described above. A QIAGEN OneStep RT-PCR Kit was used to amplify cDNAs. For *Lm*TER amplification, we used three combinations of primers a+d, e+f, and c+d (see Table S1). The primers Forward - (RT-PCR control) and Reverse - (RT-PCR control) were used to amplify *L. major* histone H2A cDNA fragment (∼150 bp), used as a control (see Table S1). Other control reactions included amplification of both cDNAs in the absence of reverse transcriptase (Superscript II, Life Technologies), in the absence of RNA, or using RNA obtained from pre-immune serum IP extracts.

## Results

### *In silico* identification of the putative TER locus within *Leishmania* genomes

We searched the five publically available *Leishmania* genomes with all six iterations of two copies of the telomeric hexamer repeat (TTAGGG) using BLASTn [24] (see Methods), based on the recently described *T. brucei* TER template, which contains an 11-nt sequence complementary to the telomeric 3′ overhang sequence [9], [21], [22], and the cloned telomeric terminus of *L. major* Friedlin [36] although the exact nature of the overhang varies between species [6], [25]. In *L. major* (the species with the best assembled genome), after running an *ad-hoc* PERL script to eliminate BLAST hits within the telomeres, only two non-telomeric loci showed hits (Table 1). Five out of six queries matched the same region on chromosome 28, while one (query_4) showed a unique match on chromosome 25. However, only the latter (between the *LmjF.25.0860* and *LmjF.25.0870* protein-coding genes) showed matches at syntenic loci in all four other *Leishmania* species (Table 2). Thus, we concluded that this region contains the putative *TER* locus in *Leishmania*.

*LmjF.25.0870* is orthologous to *Tb927.11.0850*, which is the second gene downstream of the *TER* locus in *T. brucei*, so we explored the synteny surrounding the putative *TER* locus in the genomes of three different trypanosomatid genera by using the ACT tool [26] to visualize tBLASTx comparisons of chr25 from *L. major* and *L. mexicana*, chr28 from *Crithidia fasciculata* and chr11 from *T. brucei* (Figure 1). While *L. major* and *L. mexicana* showed perfect synteny throughout the entire locus, *C. fasciculata* and *T. brucei* contained an additional protein-coding gene (*CfaC1.28.1200* and *Tb927.11.840*) immediately downstream of the *TER* locus. Conversely, *T. brucei* appeared to lack the three protein-coding genes immediately upstream of the *TER* locus in the other species, but the synteny resumed more 5′ at the *Tb927.11.0810* gene. These results support previous findings that *Crithidia* is evolutionarily closer to *Leishmania* than to *Trypanosoma* [37], [38].

**Figure 2:**
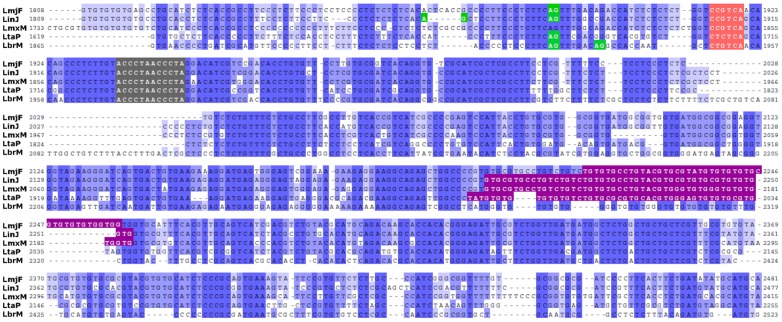
Global multiple alignment of the intercoding region on chromosome 25 where *LeishTER* is located. Coordinates are relative to the first base after the STOP codon of the respective CDSs: *L. major* (*LmjF.25.0860*), *L. infantum* (*LinJ.25.0890*), *L. mexicana* (*LmxM.25.0860*), *L. tarentolae* (*LtaP25.0910*) and *L. braziliensis* (*LbrM.25.0740*). The differently shaded colored regions represent the 12 nt template sequence (gray), 5′-C[C/T]GTCA-3′ motif that is part of the template boundary element (TBE) (pink), snoRNA domains found by the RNAspace webserver [39] (magenta, the one at position 2216–2259 in LmjF and aligned to other three species is part of snoU90 (or scaRNA7), which is a C/D box snoRNA) and splice acceptor sites (green) detected by RNA-seq (provided by Myler lab and deposited on tritrypdb.org for *L. major*). ClustalW [27] and Jalview [28] were used to align and visualize this locus, respectively. The complete alignment of the whole intercoding region is provided on Figure S3.

Multiple sequence alignment of all five *Leishmania* genomes revealed a high degree of sequence conservation throughout most of the 4.5-kb inter-CDS region containing the *TER* gene (see Figure S3), but especially surrounding the putative TER template sequence (Figure 2). Searches for non-coding RNA domains using the RNAspace webserver platform [39] revealed a∼45 nt-long small nucleolar (sno) RNA domain ∼300 nt downstream of the putative TER template sequence in four species (LmjF, LinJ, LmxM and LtaP) that best matches snoRNAU90 (or scaRNA7), a C/D box snoRNA found in human Cajal bodies [40].

### Characterization of the *Lm*TER transcript

**Figure 3:**
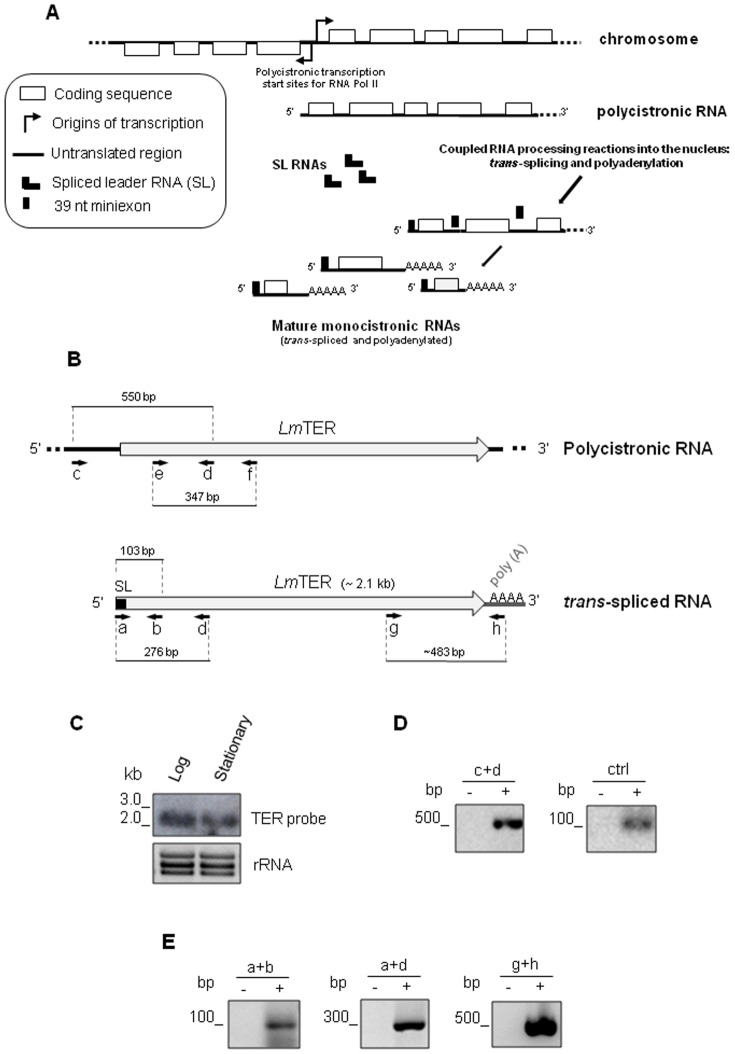
*LmTER* gene is processed by *trans*-splicing. A) Schematic representation of the general process of mature RNA synthesis in trypanosomatids adapted from Requena, J.M. (2011) [69]. After polycistronic transcription of the DNA, RNAs are individualized into monocistronic mature units through two coupled processing reactions: *trans*-splicing and polyadenylation. The former occurs at the 5′ end of the downstream gene and consists on the addition of a capped 39 nt miniexon sequence from the SL RNA, while the latter takes place at the 3′ end of the upstream gene for the poly(A) tail generation, similar to what happens on higher eukaryotes RNAs. B) A diagram showing the *LmTER* non-coding transcript, on its immature (within the polycistronic RNA precursor) and mature (*trans*-spliced and polyadenylated) forms. a, b, c, d, g and h indicate the corresponding positions of primers used in the RT-PCR and RAcE reactions. The combination of primers e and f was used to generate the northern TER probe. The expected sizes of each amplicon are denoted. C) Total RNA (10 µg), from parasite in the logarithmic and stationary phases were separated on a 1.5% agarose/2.0 M formaldehyde gel, and the northern blot was probed with a TER specific-probe, which was generated using the primers e+f. Bottom, ethidium bromide-stained RNA gel showing rRNA, served as a loading control. D) RT-PCR using primers c+d detected a band of ∼550 bp which is indicative of either a polycistronic pre-mRNA or a longer transcript, possibly the TER precursor. An amplicon of ∼150 bp from Histone H2A transcript was detected as control (ctrl). E) 5′-560 Spliced form of *Lm*TER confirmed by 5′RAcE-PCR using the following primer pairs: a+b, a+d. The 3′ end of *Lm*TER containing the polyA tail was amplified by 3′RAcE-PCR using primers g+h. See Table S1 for a complete description of primers. Control reactions (-) were done in the presence of Taq polymerase only.

Northern analysis of RNA extracted from *L. major* promastigotes using a 347-nt probe specific for the putative *LmTER* locus, identified a major transcript of ∼2,100 nt (Figures 3B and 3C) and a similar result was obtained for *L. amazonensis* (Figure S1). We also found that both *LmTER* and *LaTER* RNAs were expressed at similar levels in the logarithmic and stationary growth phase (Figure 3C and Figure S1). Taking into account that trypanosomatid ncRNAs transcribed by RNA polymerase II, such as snoRNAs and *Tb*TER, are *trans-*spliced and polyadenylated (Figure 3A) [21], [22], [41], we performed both 5′ and 3′ rapid amplification of cDNA ends (RAcE) PCR to respectively identify the SL site at the 5′ end and the polyA tail at the 3′end of the *Lm*TER transcript. First strand cDNA synthesis with internal primers b or d (Figure 3B and Table S1) followed by second strand synthesis using the SL-specific primer a (Figure 3B and Table S1) revealed products of 103 bp and 276 bp for the 5′RAcEs (Figures 3B and 3E). The 3′RAcE revealed a product of ∼500 bp (Figures 3B and 3E), using oligo dT-specific primer h for the first strand cDNA synthesis and an internal primer g for the second strand (Figure 3B and Table S1). Sequencing of the 5′RAcE PCR products (Figure S2) mapped the 5′ SL site to the position 333,307 on *L. major* chromosome 25 (Figure 2), while the 3′RAcE product size is indicative of a polyA site at position 335,419 on the same chromosome. These results are consistent with SL and PolyA sites previously assigned to this genomic locus by RNA-seq experiments (tritrypdb.org). On the other hand, RT-PCR using primers c+d, showed a 550 bp cDNA product (Figure 3D), presumably representing the longer polycistronic transcript (Figures 3A and 3B). Automated sequencing of this cDNA product confirmed they came from the targeted locus (data not shown).

### *Lm*TER forms a ribonucleoprotein complex with LmTERT

**Figure 4:**
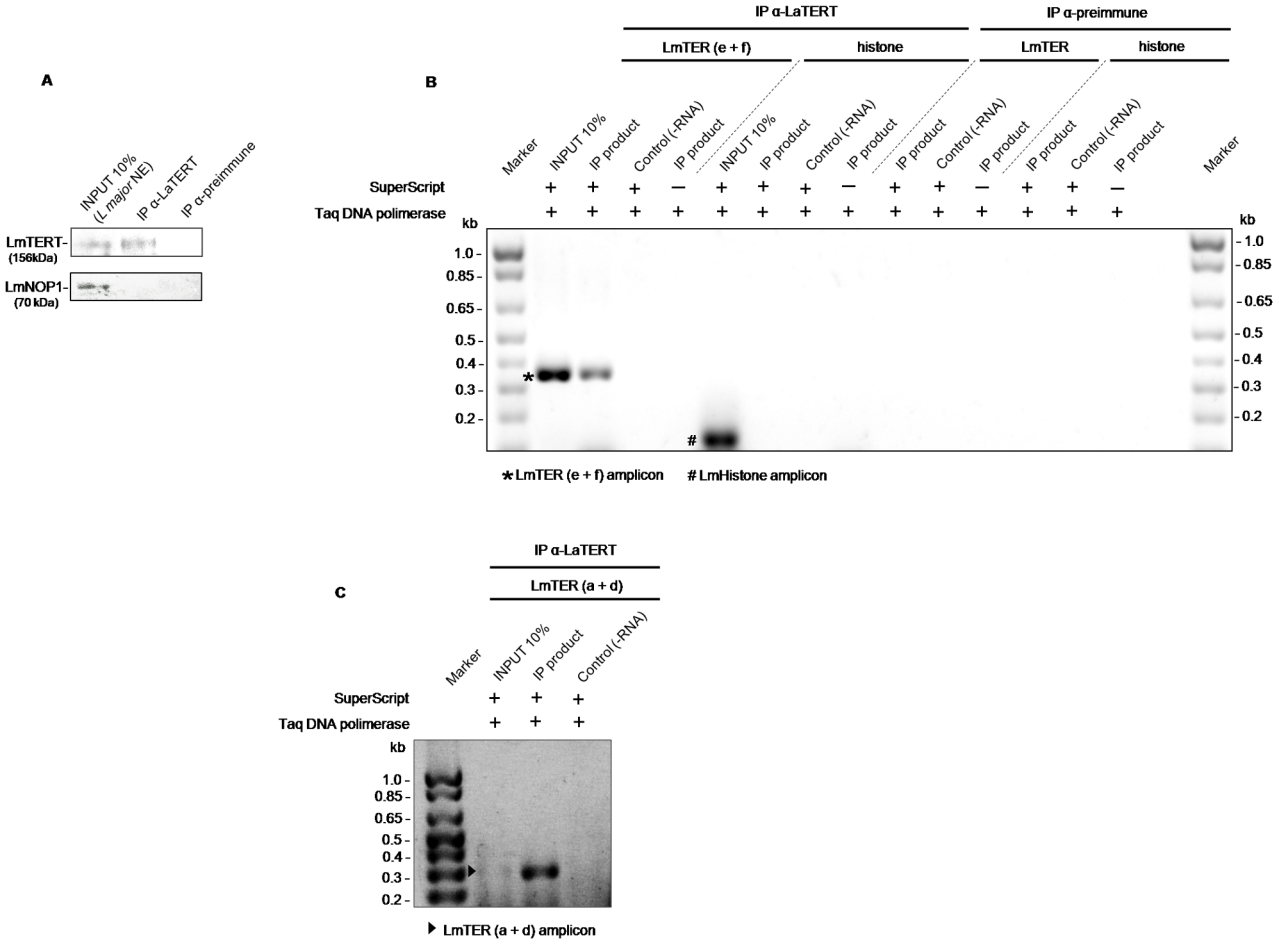
*Lm*TER is amplified from extracts immunoprecipitated with anti-LaTERT serum. A) Ten percent of a *L. major* nuclear extract (*L. major* NE) used as input in the IP assay and a rabbit anti-LaTERT IP eluate (10%) were fractionated in a SDS-PAGE gel and assayed in a western blot revealed with mouse anti-LaTERT (upper panel) and rabbit anti-LmNop1 (bottom panel). B) Ethidium bromide-stained agarose gel of RT-PCR of *Lm*TER and *L. major* histone cDNAs obtained from RNAs isolated from the *L. major* nuclear protein extract (input) and from *L. major* nuclear extract immunoprecipitated (IP eluate) with anti-LaTERT (IP product). *Lm*TER was amplified with primers e+f and histone H2A was amplified as a RT-PCR control. Rabbit pre-immune serum was used as an IP control. C) 5′-Spliced form of *Lm*TER was amplified by RAcE-PCR from RNAs isolated from *L. major* nuclear extract (input) and from an anti-LaTERT IP eluate using primers a+d.

Immunoprecipitation assays were conducted to identify the *Lm*TER transcript that formed a complex with the telomerase reverse transcriptase (TERT) protein component in protein parasite extracts. Western blot analysis of *L. major* S100 nuclear extracts immunoprecipitated with rabbit anti-LaTERT serum confirmed the presence of LmTERT (MW 156kDa) in both parasite nuclear extract (input 10%) and IP eluate (Figure 4A upper panel), while the LmNOP1 (MW ∼70 kDa) control was present only in the parasite nuclear extract (input 10%) (Figure 4A bottom panel) and thus, was not immunoprecipitated by anti-LaTERT, confirming anti-LaTERT specificity.

Total RNA isolated from the IP eluates shown in figure 4A was used to amplify fragments of the *Lm*TER transcript using RT-PCR. The results of RT-PCR using primer pairs a+d and e+f amplified products of expected sizes, respectively ∼276 bp and ∼347 bp (Figures 4B and 4C). We were not able to amplify the 3′end of the IP *Lm*TER using primers g+h (data not shown), thus, we cannot certify that the *Lm*TER in complex with LmTERT is polyadenylated. In addition, the reaction with primers c+d did not amplify any product from the IP extract (data not shown), probably because primer c is located upstream of the expected SL site for the mature *Lm*TER transcript. This result gives support to the evidence that there might be only one SL site for the mature *Lm*TER (SL at position 333,307 on chromosome 25, as mentioned above) and that only the mature transcript co-immunoprecipitates with the LmTERT component. The pre-immune sera did not immunoprecipitate any of the tested proteins from the parasite extract and thus, no RNA was isolated from these reactions. As a control, *Leishmania* histone H2A was amplified as a∼150 bp amplicon using specific primers (Table S1) only in the input sample, demonstrating the specificity of the IP assay (Figure 4B).

Together, these results confirm that the SL sequence-containing mature *Lm*TER RNA is part of the *Leishmania major* telomerase ribonucleoprotein complex.

### *Lm*TER co-localizes with LmTERT in promastigotes of *L. major*

**Figure 5:**
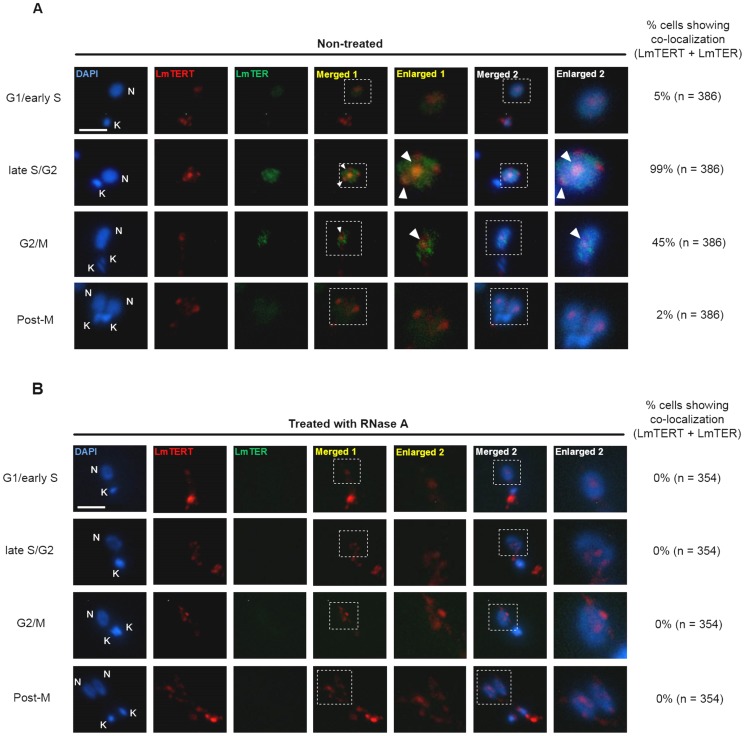
*Lm*TER co-localizes with LmTERT in late S/G2 phase. RNA FISH coupled with IIF with anti-LaTERT serum. Cells were analyzed throughout the *L. major* cell cycle. “Merged 1” combines images from LmTERT and *Lm*TER. “Merged 2” combines all images. Co-localization foci (white arrows) containing *Lm*TER and LmTERT occur mainly at late S/G2 phases (A). In cells treated with RNase A (B), no RNA hybridization signal or co-localization was detected, indicating that the results shown in (A) correspond to *Lm*TER and LmTERT co-localizing at the same foci. DAPI (blue) was used to stain DNA in kinetoplast (K) and nucleus (N). These figures contain representative cells of a series of images captured randomly to avoid bias. Scale bar represents 2 µm.

RNA Fluorescent *In Situ* Hybridization (FISH) of *L. major* promastigotes coupled with indirect immunofluorescence (IIF) was also performed and confirmed that *Lm*TER co-localizes with LmTERT. A TelG fluorescein-conjugated PNA probe containing three telomeric hexameric repeats co-localized with IIF signal obtained with rabbit anti–LaTERT serum (Figure 5A). Although there were several clusters of *Lm*TER in most cells, only a few co-localized with LmTERT. These additional clusters may be due to hybridization between the telomeric PNA probe and an immature form of *Lm*TER that is not associated with the LmTERT ribonucleoprotein complex. The FISH signal was absent in cells pretreated with RNase A, indicating that the probe was specific for *Lm*TER RNA and did not cross-hybridize with telomeric DNA (Figure 5B). *Lm*TER was most abundant in late S/G2 and M phase of the parasite cell cycle, coincident with the timing of telomere replication in *Leishmania* promastigotes (da Silva & Cano, unpublished data) and in budding yeast [42].

### *Lm*TER putative secondary structure

**Figure 6:**
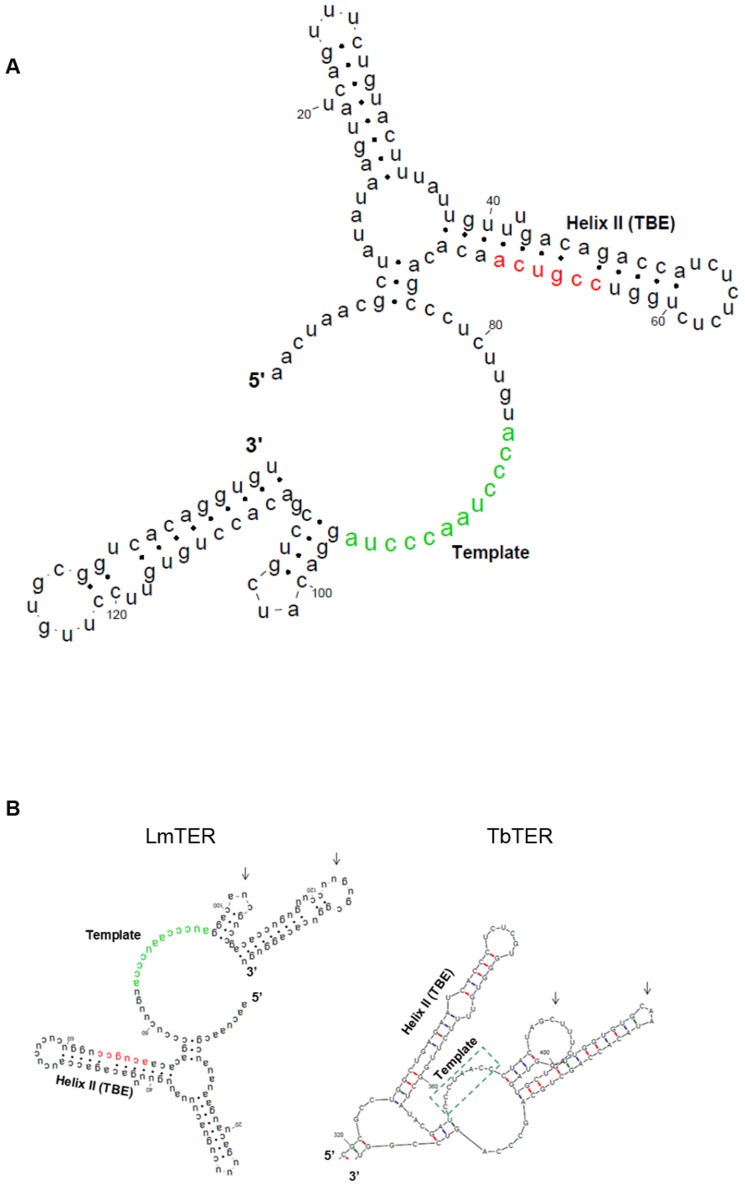
The predicted secondary structure model of *Lm*TER. A) Proposed secondary structure (mfold - default parameters, followed by visualization through RNAviz editor [70]) obtained from the first 139 nucleotides of *Lm*TER (39 nt SL sequence from the 5′ cap processing plus 100 nt from the beginning of the gene in the genome). This folding prediction in that particular region led us to infer the existence of two crucial structured domains already detected in all other TERs reported hitherto: (*i*) Helix II, containing a CCGUCA motif (red) at its proximal 3′ end, which is implicated in proper template boundary definition in *Tetrahymena thermophila*; and (*ii*) the single-stranded template sequence (green). B) The *Lm*TER structure in A was turned upside-down and compared to the ∼100 nt surrounding template *Tb*TER structure (the *Tb*TER sequence used in this analysis and also the default parameters to run the mfold program were identical to those indicated by Gupta and colleagues, 2013). The dashed green box represents the template sequence in *Tb*TER. Arrows indicate similar shape of hairpin structures immediately downstream of the template on both TERs.

A putative secondary structure of *Lm*TER was obtained by using its first 5′ 139 nt as input to the mfold tool [30] (Figure 6A). Some structural features of *Lm*TER that are conserved in all TERs already described are signaled in Figure 6A, such as the single-stranded template sequence highlighted in green (5′ ACCCTAACCCTA 3′) at position 85, Helix II or TBE (Template Boundary Element), comprising the conserved short motif 5′-CCGUCA-3′ (highlighted in red), and the GC base pairing both at the proximal end of Helix II.

A comparison between our predicted initial 139 nt *Lm*TER structure with the one encompassing ∼100 nt surrounding template sequence from *Tb*TER is shown in Figure 6B. Here we can observe that despite the differences, for example in the position of the template sequence between *Lm*TER and *Tb*TER, we identified similar structural features such as the TBE and two additional hairpins right downstream of the template, which maintain the general shape conservation and geometry in both molecules.

## Discussion

In contrast to protein-coding DNA, non-coding RNA genes are prone to several non-lethal mutations related to no discernable phenotype [43] principally due to compensatory nucleotide substitutions, which maintain the secondary structure of the final RNA molecule despite the modifications in the primary gene sequence. This assertion fits on what we observed for the *LeishTER* gene. With the exception of the TER template sequence and short stretches randomly aligned, no overall sequence similarity was found between telomerase RNA genes from the *Leishmania* and *Trypanosoma* genera (*LeishTER* and *TbTER*). More than two decades ago, in a study of ciliate TER structure, Romero and Blackburn observed high sequence content divergence between the TER genes of *Tetrahymena thermophila* and *Euplotes crassus* (holotrichous and hypotrichous ciliates, respectively), which prevented them from either aligning the sequences or identifying common structural elements with confidence among those distinct genera [44]. This special ncRNA feature highlights the need of applying a non-trivial computational method to capture the *LeishTER* gene on focus in this study. We have successfully developed our own *in silico* approach to scan *LeishTER* candidates throughout *Leishmania* genomes based on the 12 nt TER template sequence previously assigned to *L. amazonensis* [6]. The TER template sequence is a crucial component of the TERT holoenzyme, possessing the antisense orientation of the telomeric hexamer repeat (THR) and guiding the telomerase reverse transcriptase activity on the elongation of telomeres [9], [21], [45]. After filtering out all THR hits that fell onto *Leishmania* telomeres, two non-telomeric loci showed hits in *L. major* genome (Table 1) and only one (on chromosome 25 between the *LmjF.25.0860* and *LmjF.25.0870* protein-coding genes) presented matches at syntenic loci in all *Leishmania* species studied herein (Table 2). This region was assigned by us as containing the putative *TER* locus in *Leishmania*.

Trypanosomatids share a remarkable degree of synteny between their genomes [46]. Therefore, we took advantage of this evolutionarily trait to perform comparative genomics analyses to reliably identify the *LeishTER* candidate locus by *in silico* methods. We verified that *LmjF.25.0870* (the gene immediately downstream of *LmTER*) is orthologous to *Tb927.11.0850*, which is the second gene downstream of the *TbTER* gene mapped on chr11 from *T. brucei* [21], [22]. Exploring the synteny on the TER locus between three different trypanosomatid genera, it was possible to detect a lack of conservation on the TER-flanking protein-coding genes between *Trypanosoma* and *Leishmania* species, with the synteny resuming more 5′ at the *Tb927.11.0810* and *LmjF.25.0830* genes, and 3′ at *Tb927.11.0850* and *LmjF.25.0870* (see results and Figure 1). We believe that this disruption of synteny for some protein-coding genes was the main adverse factor that prevented others from using the *TbTER* location to easily map the *TER* gene within *Leishmania* genomes. This assessment shows us that trypanosomatid TER non-coding RNA gene appears to be maintained by selective pressure within a synteny-disturbed locus, which might indicate its functional relevance. It is also clear, by assessing the loci displayed in Figure 1, that *Crithidia* genus seems to be evolutionarily closer to *Leishmania* than to *Trypanosoma*, representing a middle branch between the latter genera, corroborating other studies on molecular evolution of trypanosomatids [37], [38]. It is noteworthy that the *Cf*TER transcript has not yet been experimentally characterized. Therefore, similar molecular approaches performed in this work and on both *Tb*TER publications [21], [22] need to be addressed to validate the *CfTER* gene.

By thoroughly analyzing the entire *LeishTER*-containing intercoding region (>4.5 kb) within the chromosome 25 from five *Leishmania* species (LmjF, LinJ, LmxM, LtaP and LbrM), we discovered a series of interesting features: (i) The putative 12 nt template sequence was perfectly aligned on all sequences in the global multiple alignment comparison (Figure 2 and Fig. S3), although it has been previously reported that the telomeric terminal overhang is different in length and at the 3' end nucleotides in *L. amazonensis* and *L. donovani/L. major* [6], [25], [36]. A possible explanation for this situation can be the occurrence of a non-conserved resection process that couples the removal of the RNA primer after DNA replication and the action of an exonuclease to generate longer 3′G-overhangs that are substrates for telomerase elongation. This is a very complex event that was poorly described in eukaryotic models and is still unknown in *Leishmania* spp. [47]. (ii) Considering that non-protein-coding regions are poorly conserved among the genomes of *Leishmania* species from different subgenera [48], we found the following overall identities among the *LeishTER*-containing intercoding regions: averages of 80.7% between species from *Leishmania (Leishmania)* subgenus (LmjF, LinJ and LmxM), 48.5% between *Leishmania (Sauroleishmania) tarentolae* and *Leishmania (Leishmania)* spp., and 49.6% between *Leishmania (Viannia) braziliensis* and *Leishmania (Leishmania)* spp. (Figure S3). It is also noteworthy that an independent and previously published large-scale mapping of conserved intercoding sequences (CICS) on the LmjF, LinJ and LbrM genomes has reported at least four short CICS within the putative *LeishTER* genes, one of which is 41 nt-long (LeishCICS-s8786) and encompasses the 12 nt TER template sequence [49], suggesting that it might be a conserved functional domain of the *Leish*TER ncRNA molecules. (iii) A novel ∼3.6 kb gene (*LmjF.25.T0865*) was recently mapped within the *LmTER*-containing intercoding region and overlaps to the *LmTER* gene. This discovery was made together with 1,883 other new genes that were identified by a polyA-captured RNA-seq assessment in *L. major* [50]. Rastrojo and colleagues (2013) have performed no individual functional characterization on any of those novel annotated transcripts and claimed they should be considered ncRNAs until shown to be otherwise. Due to the experimental results we have gotten for *Lm*TER (Figures 3 and 4), and taking into account that there are several SL sites within the inter-CDSs region between *LmjF.25.0860* and *LmjF.25.0870* protein-coding genes (RNA-seq data info provided on tritrypdb.org), we believe that *LmjF.25.T0865* gene might reflect either a junction of two or more transcripts or a longer precursor of *LmTER*. (iv) One ∼45 nt-long small nucleolar RNA domain was detected right downstream of the TER template sequence within the transcripts of four species (LmjF, LinJ, LmxM and LtaP) (Figures 2 and Figure S3, shaded in magenta). It is part of the snoU90 (or scaRNA7), which is a C/D box snoRNA found in human Cajal bodies [40]. This finding partially corroborates the results of Gupta and colleagues (2013), which suggested that *Tb*TER is a member of the C/D box class of snoRNAs due to its affinity selection by epitope tagged TERT and SNU13 (a C/D snoRNA-binding protein), but not by NHP2 (a protein that binds H/ACA snoRNAs) [22]. Despite these findings, whether both *Tb*TER and *Leish*TER act with a function other than the TERT-associated one remains an open question.

As mentioned earlier in this section, Northern blot analyses identified a∼2,100 nt *Lm*TER transcript, but its precursor was not detected (Figure 3C) probably because the mature *Lm*TER might be much more abundant in the total RNA extract. Thus, low levels of *Lm*TER precursor should only be detected by a high sensitive method like RT-PCR (Figure 3D). Molecular assays supporting this hypothesis were very well conceived by the trypanosomatid research community within the past 2–3 decades [54], [55]. For example, studies with the tandem array of tubulin genes revealed that polycistronic pre-mRNA precursors are visualized on Northern blots only when the transcripts accumulate after *trans*-splicing blockage by heat shock [54], also polycistronic HSP7O pre-mRNA appeared to be rare in the nascent RNA population, possibly because of rapid processing of the nascent RNA in the intergenic region by cleavage for *trans*-splicing and polyadenylation [55].

In addition, *Leish*TER and the *Plasmodium falciparum* TER (∼2,200 nt, [51]) might be the longest protozoa TERs described so far, exceeding the lengths of the most commonly studied TERs in other organisms such as the ciliate *Tetrahymena termophila* (159 nt, AF399707.1), the budding yeast *Saccharomyces cerevisiae* (∼1,300 nt, AM296228.1), the vertebrates *Mus musculus* (590 nt, MMU33831) and *Homo sapiens* (450 nt, AF047386.1), and the closely related species *T. brucei* (993 nt) [22], [52]. The longest TER described so far is from *Candida glabrata*, a parasitic species close to *S. cerevisiae* that has a 2.7 kb-long TER [53]. Moreover, similar levels of *Lm*TER mature transcript were detected during exponential promastigote growth (logarithmic and stationary phases), suggesting that the non-replicative forms of the parasite contains the same *Lm*TER levels as the replicative ones. Although we do not have an experimental answer to this result, the most probable explanation is that *Leish*TER is a highly stable transcript, which is not degraded during parasite growth, as identical results were also obtained with *L. amazonensis* TER (Figure S1 and data not shown).

The reverse transcription polymerase chain reactions (RT-PCRs) of total RNA succeeded and the sequencing of these RT-PCR products (Figure 3E) allowed us to confirm the *Lm*TER sequence (Fig. S2) and strongly indicated that *Lm*TER has a 5′ SL cap added by *trans*-splicing at position 333,307 and a possible polyA tail added at position 335,419 from *L. major* chromosome 25 (Figures 2 and 3E), corroborating RNA-seq evidence for SL and polyA sites on this genomic region (tritrypdb.org).

The association of the *trans*-spliced *Lm*TER form with LmTERT was first evidenced using both RT-PCR and 5′ RAcE both primed with different pairs of primers (Figures 4B and 4C) resembling its *Tb*TER counterpart [21]. However, it was not possible to certify whether the *Lm*TER transcript associated with LmTERT was terminated by the addition of a polyA tail because we could not amplified any product from the IP eluate by 3′ RAcE (data not shown), suggesting that the mature *Lm*TER that associates with LmTERT is not polyadenylated. Our hypothesis to explain this result relies on the fact that different mechanisms have evolved for telomerase RNA 3′ end formation. Well-studied TERs (e.g., budding and fission yeasts and human), which are also RNA pol II-transcribed, have their 3′ end processed at sites located upstream to the mapped polyadenylation sites. Therefore, after cleavage reaction(s), they are polyA(-) transcripts [56]–[61]. Non-canonical 3′ end processing mechanisms, such as cleavage by RNase P, are able to process RNA pol II nascent transcripts to generate their mature 3′ ends despite the presence of nearby polyadenylation signals. It seems that a significant fraction (>25%) of long transcripts present in cells, which includes the telomerase RNAs, lack a classical polyA tail and that the selection of a proper 3′ end cleavage site represents an important step, not only for the post-transcriptional regulation of gene expression, but also to generate the mature 3′ ends of these transcripts via multiple mechanisms, (reviewed [62]). For example, in both *Schizosaccharomyces pombe* and *Saccharomyces cerevisiae*, the functional/mature telomerase RNAs have their 3′ end processed by a specific spliceosomal cleavage mechanism and by transcription termination factors such as Nrd1 and Nab3, respectively [58], [60], [61], [63]. We attempted without any success to find neither Nrd1/Nab3 homolog sequences in *Leishmania spp.* and *T. brucei* genomes nor short conserved *cis*-elements/motifs within our putative *Lm*TER 3′ end boundary region that would be recognized by those yeast factors (data not shown). Thus, further investigations are required to determine whether the *Lm*TER mature transcript also terminates in a non-canonical 3′ end site.

Co-localization assays using RNA-FISH coupled with IIF using a specific anti-telomerase serum, confirmed that *Lm*TER and LmTERT partially associate principally at late S-G2 phase of the promastigote cell cycle, coinciding with the timing of parasite telomere replication (da Silva MS and Cano, unpublished data). The access of human and yeast TERs to telomerase and their substrates are regulated as a function of the cell cycle. In humans, hTER and hTERT are found in distinct nuclear foci throughout most of the cell cycle. Only during S phase, hTER, which is mainly retained in Cajal bodies, moves with TERT in the direction of telomeres. In yeast, in contrast, the mature TER (TLC1) is first exported to the cytoplasm to assemble with the telomerase holoenzyme, and then the entire RNP complex re-enters the nucleus and only in S phase TER co-localizes with a few telomeres; and like in humans, yeast TER accumulation does not also require assembly with TERT (reviewed in [61]). Despite the clear evolutionary divergence among trypanosomatids, yeast and vertebrates, it is possible that they all share common features of the telomerase RNP biogenesis pathway and regulation.

Relying on the results of the RT-PCR from the TERT-IP nuclear extracts, where we used the SL sequence as the forward primer (Figure 4C), we found that the splice site for the TERT-interacting mature *Lm*TER is near the template sequence (as ascribed on Figure 2 and above discussed). In contrast to *Tb*TER, which was reported to present the template sequence located far from the 5′end of TER (position 370), *Lm*TER template is fairly close to its 5′ end (position 85) (Fig. 6), similarly to ciliates and vertebrate TERs [64]. Although we could not propose a secondary structure for the entire *Lm*TER RNA molecule, due to its remarkable length (>2 kb) and the marked drop in computational prediction accuracy as the sequence length increases [65], we were able to detect, by using the first 5′ 139 nt from *Lm*TER as input to the mfold tool, some *bona fide* structures, such as the single-stranded region encompassing the template sequence and Helix II [30]. Similar results regarding these two *bona fide* structures were also retrieved when we attempted to run RNAalifold [29], using a multiple alignment of all *Leish*TERs studied herein, and then applying the constraints from the consensus structure on the individual TER modeling by executing mfold [30] (data not shown). Figure 6A depicts both the single-stranded template sequence and Helix II, which harbors elements required for the proper template boundary definition (TBE) [66]–[68]. The conserved short motif 5′-CCGUCA-3′, though slightly different from the one in *Tetrahymena* (5′-CUGUCA-3′), is also found at the proximal 3′ end of Helix II in *Leishmania* TERs, as it is in ciliates [68]. Notably, we observed the exact match 5′-CUGUCA-3′ in *L. braziliensis* TER (Figure 2 and Figure S3, 21 nt upstream of the template sequence). More important, the GC base pairing at the proximal end of Helix II, which is essential for proper template boundary definition and required for binding of the *Tetrahymena* telomerase reverse transcriptase (TERT) [68], is also present in both *Plasmodium falciparum* TER (*Pf*TER) and *Leish*TERs TBEs, although both RNA secondary structures have yet to be experimentally validated.

The comparison between the generated *Lm*TER and *Tb*TER structures (Figure 6B) showed that despite the huge differences, for example in the template sequence location within both TERs, we identified similar structural features (e.g. same shape and geometry of two stem-loops right downstream of the template) that might be suggestive of common interaction pathways with their respective TERT partners and, consequently, similar functionality in the holoenzyme complex.

In this manuscript we have identified and partially characterized *Leish*TER, a long non-coding RNA that preserves some conserved features and also presents unique ones that categorize it as a strong candidate for the *Leishmania* TER component. Despite its similarities with the recently described *Tb*TER component, further structure-driven studies need to be addressed to unravel the biochemical and biophysical details of the whole *Leishmania* TERT core complex and the importance of telomerase biogenesis for parasite cell survival and homeostasis.

## Supporting Information

Figure S1 Molecular validation of *L. amazonensis* TER candidate. A) 5′ Spliced form of *La*TER was confirmed by RAcE-PCR using primers a+b; arrows indicate nonspecific amplified bands. The putative 3′ end of *La*TER containing the polyA tail was also confirmed by RAcE-PCR using primers g+h. *La*TER was detected from the polycistron using primers c+d. Histone H2A was used as control (ctrl). B) Total RNA (10 µg) from parasites in the logarithmic and stationary phases of growth were separated on a 1.5% agarose/2.0 M formaldehyde gel, and the blot was probed with a *Lm*TER-specific-probe, which was generated using the combination of primers e+f. Bottom, ethidium bromide-stained RNA gel showing rRNA as the loading control. The primers used in assays shown in A) and B) are the same used in Figures 3 and 4 and are listed in Table S1.
(TIF)

Figure S2 *Lm*TER undergoes *trans*-splicing. cDNA prepared from wild-type *L. major* and *L. amazonensis* cells were cloned into the TOPO-TA vector (Invitrogen). The pre-*Leish*TER sequence was amplified using a sense SL RNA primer and an internal reverse primer from the *Lm*TER sequence (as shown in Figure 3). The positions of the spliced leader (SL) (blue), Helix II structure (red), and template (green) are depicted.
(TIF)

Figure S3 Complete global multiple alignment and identity matrix of the entire intercoding region of *Leish*TER locus. Coordinates are relative to the first base after the stop codon of the respective CDSs: *L. major* (*LmjF.25.0860*), *L. infantum* (*LinJ.25.0890*), *L. mexicana* (*LmxM.25.0860*), *L. tarentolae* (*LtaP25.0910*) and *L. braziliensis* (*LbrM.25.0740*). The differently shaded colored regions represent the 12 nt template sequence (gray), 5′-C[C/T]GTCA-3′ motif that is part of the template boundary element (TBE) (pink), snoRNA domains reported by the RNAspace webserver (magenta), splice acceptor sites upstream of the template sequence (green) and polyA sites downstream of the template (red) identified by RNA-seq (provided by Myler lab and deposited on tritrypdb.org for *L. major*). ClustalW run locally and on the web, as well as Jalview, were used to align the sequences, build the identity matrix and visualize the alignment, respectively.
(PDF)

Table S1 List of primers.
(DOC)
