# Supplementary figures and images for: The Putative Leishmania Telomerase RNA (LeishTER) Undergoes Trans-Splicing and Contains a Conserved Template Sequence
Source: PLoS One. 2014 Nov 12;9(11):e112061. doi: 10.1371/journal.pone.0112061 (PMC4229120; doi:10.1371/journal.pone.0112061)

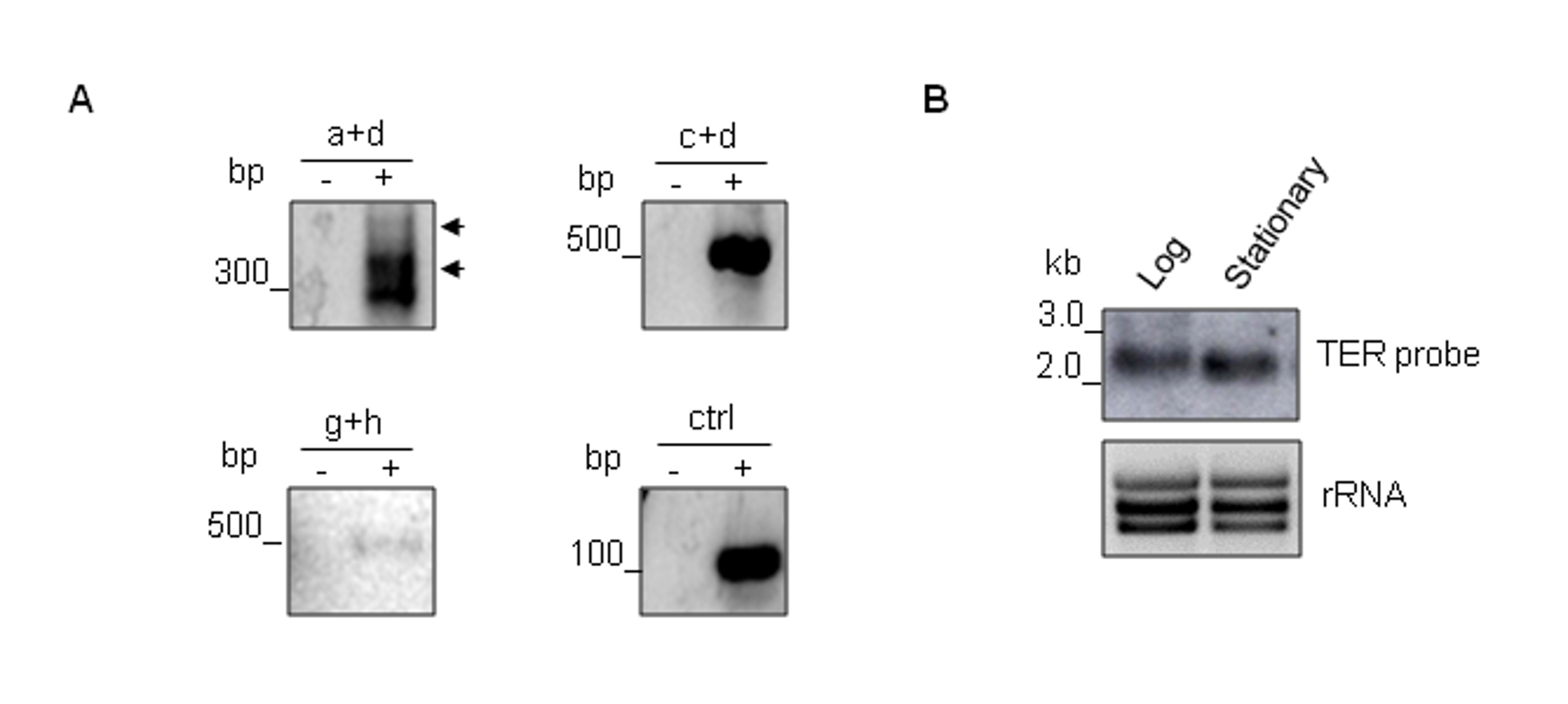

Supplement: Figure S1 — Molecular validation of L. amazonensis TER candidate. A) 5′ Spliced form of LaTER was confirmed by RAcE-PCR using primers a+b; arrows indicate nonspecific amplified bands. The putative 3′ end of LaTER containing the polyA tail was also confirmed by RAcE-PCR using primers g+h. LaTER was detected from the polycistron using primers c+d. Histone H2A was used as control (ctrl). B) Total RNA (10 µg) from parasites in the logarithmic and stationary phases of growth were separated on a 1.5% agarose/2.0 M formaldehyde gel, and the blot was probed with a LmTER-specific-probe, which was generated using the combination of primers e+f. Bottom, ethidium bromide-stained RNA gel showing rRNA as the loading control. The primers used in assays shown in A) and B) are the same used in Figures 3 and 4 and are listed in Table S1. (TIF) [file pone.0112061.s001.tif]

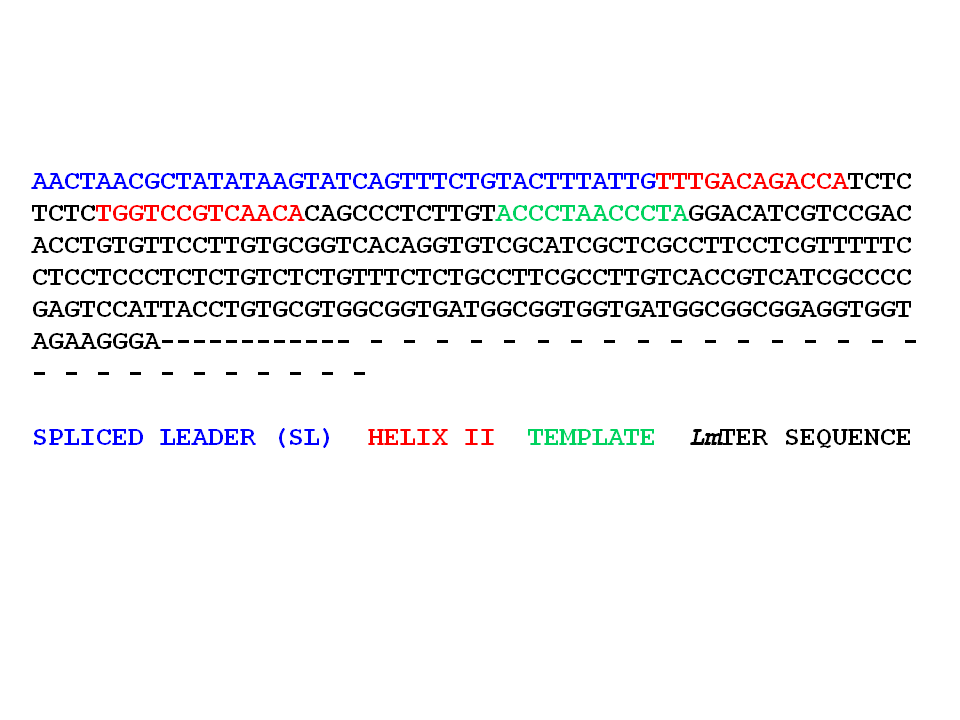

Supplement: Figure S2 — LmTER undergoes trans-splicing. cDNA prepared from wild-type L. major and L. amazonensis cells were cloned into the TOPO-TA vector (Invitrogen). The pre-LeishTER sequence was amplified using a sense SL RNA primer and an internal reverse primer from the LmTER sequence (as shown in Figure 3). The positions of the spliced leader (SL) (blue), Helix II structure (red), and template (green) are depicted. (TIF) [file pone.0112061.s002.tif]
