# Supplementary material for: The Putative Leishmania Telomerase RNA (LeishTER) Undergoes Trans-Splicing and Contains a Conserved Template Sequence
Source: PLoS One. 2014 Nov 12;9(11):e112061. doi: 10.1371/journal.pone.0112061 (PMC4229120; doi:10.1371/journal.pone.0112061)
Supplement: Figure S3 — Complete global multiple alignment and identity matrix of the entire intercoding region of LeishTER locus. Coordinates are relative to the first base after the stop codon of the respective CDSs: L. major (LmjF.25.0860), L. infantum (LinJ.25.0890), L. mexicana (LmxM.25.0860), L. tarentolae (LtaP25.0910) and L. braziliensis (LbrM.25.0740). The differently shaded colored regions represent the 12 nt template sequence (gray), 5′-C[C/T]GTCA-3′ motif that is part of the template boundary element (TBE) (pink), snoRNA domains reported by the RNAspace webserver (magenta), splice acceptor sites upstream of the template sequence (green) and polyA sites downstream of the template (red) identified by RNA-seq (provided by Myler lab and deposited on tritrypdb.org for L. major). ClustalW run locally and on the web, as well as Jalview, were used to align the sequences, build the identity matrix and visualize the alignment, respectively. (PDF) [file pone.0112061.s003.pdf]

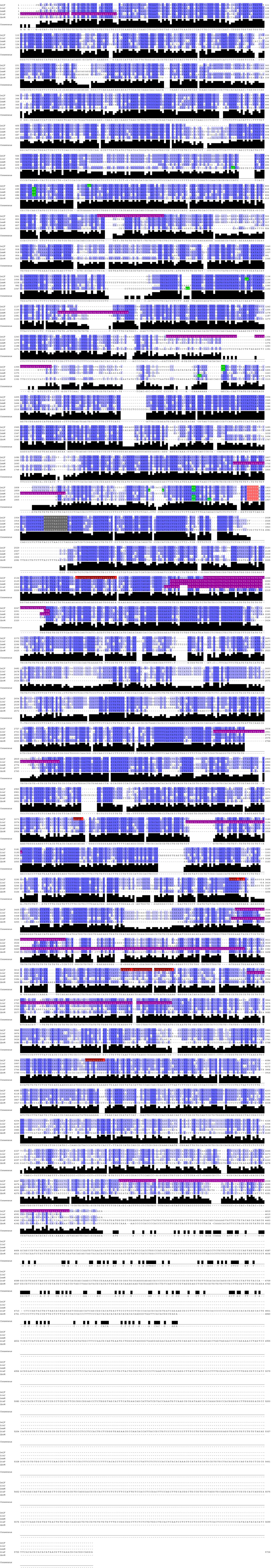

Identity matrix from the whole multiple alignment above

**LmjF**	----	85.6	78.1	48.8	50.4
**LinJ**	85.6	----	78.5	48.9	50.0
**LmxM**	78.1	78.5	----	47.7	48.3
**LtaP**	48.8	48.9	47.7	----	40.9
**LbrM**	50.4	50.0	48.3	40.9	----
