# Supplementary material for: The Putative Leishmania Telomerase RNA (LeishTER) Undergoes Trans-Splicing and Contains a Conserved Template Sequence
Source: PLoS One. 2014 Nov 12;9(11):e112061. doi: 10.1371/journal.pone.0112061 (PMC4229120; doi:10.1371/journal.pone.0112061)
Supplement: Table S1 — List of primers. (DOC) [file pone.0112061.s004.doc]

| **Primer ID** |  | **5'-3' sequence** |  | **Description** |
| --- | --- | --- | --- | --- |
| **a** |  | CGCTATATAAGTATCAGTTTCTGT |  | Forward - spliced leader sequence (5' RACE) |
| **b** |  | GATGTCCTAGGGTTAGGGTACA |  | Reverse (5' RACE) |
| **c** |  | AGAAAGGCTGGGAGCAGACG |  | Forward - (RT-PCR) |
| **d** |  | CCCTTCTACCACCTCCGC |  | Reverse - (5' RACE) |
| **e** |  | TGTACCCTAACCCTAGGACATC |  | Forward - (TER probe PCR and 3’ RACE) |
| **f** |  | CTGCAACGTGAAATGCACCA |  | Reverse - (TER probe PCR) |
| **g** |  | GCCCGTCTCGTGTGGAG |  | Forward (3’RACE) |
| **h** |  | TTTTTTTTTTTTTTTTTTTT |  | Reverse - oligo dT (3’RACE) |
| **histone 2A** |  | CTGCTGGAGCTGTCTGTGA |  | Forward - (RT-PCR control) |
| **histone 2A** |  | GTGAGACAAGGTCACGCTCT |  | Reverse - (RT-PCR control) |

**Table S1.** List of primers
